# Examinations by the Regular Board of Medical Examiners, Atlanta, Ga., April 4, 1895

**Published:** 1895-05

**Authors:** 


### EXAMINATIONS BY THE REGULAR BOARD OF MEDICAL EXAMINERS, ATLANTA, GA., APRIL 4, 1895.

### PHYSIOLOGY AND CHEMISTRY. (E. R. ANTHONY, M.D.)

Question 1. Mention the different groups of Proximate Principles.

Q. 2. Mention the different classes of Alimentary Principles.

Q. 3. Give the source, composition, and physiological action of Gastric Juice.

Q. 4. By what route do Peptones and Glucose find their way into the general circulation?

Q. 5. Give the composition and functions of Pancreatic Juice.

Q. 6. By what route do the Emulsified Fats find their way into the general circulation?

Q. 7. What is the cause of the pulse?

Q. 8. What are the factors entering into the production of the First and Second Sounds of the Heart?

Q. 9. State the changes effected in the Air by Respiration. The changes in the Blood.

Q. 10. What is the cause of the Heat of the Body?

Q. 11. What is the mean normal Temperature of the body? Give some conditions that would produce variations in this Temperature.

Q. 12. What is the function of the Cerebellum? Of the Corpora Quadrigemina?

CHEMISTRY. (E. R. ANTHONY, M.D.)

Question 1. What do you understand by the Atomic Theory?

Q. 2. What do you understand by Valency?

Q. 3. What are Acids, Bases, and Salts?

Q. 4. What is Calomel? Give formula for same.

Q. 5. What is Blue Stone? Give formula for same.

Q. 6. What is Haines's Test for Sugar in the Urine?

Q. 7. What is Heller's Test for Albumin in the Urine?

Q. 8. Give me the Antidotes for poisoning from the Caustic Alkalies. Give the Antidotes for poisoning from the Mineral Acids.

PRACTICE OF MEDICINE. (J. B. BAIRD, M.D.)

Question 1. (*a*) Give prominent symptoms, (*b*) general indications for treatment, and (*c*) appropriate means of treatment in typhoid fever.

Q. 2. Name several varieties of malarial fever. (*a*) Give symptoms of each and (*b*) proper methods of treatment.

Q. 3. Give differential diagnosis of typhoid and remittent fevers.

Q. 4. What is acute cystitis? (*a*) Give symptoms, (*b*) principal indications for treatment, and (*c*) approved means of treatment.

Q. 5. Give differential diagnosis of acute lobar pneumonia, and acute bronchitis.

Q. 6. (*a*) What is valvular disease of the heart? (*b*) Name some of the varieties (if more than one). (*c*) Give symptoms, (*d*) signs, and (*e*) appropriate means of treatment.

Q. 7. (*a*) Give diagnostic symptoms, (*b*) causes, and (*c*) treatment of iritis.

Q. 8. (*a*) State nature, (*b*) course, (*c*) termination, and (*d*) treatment of acute tonsillitis.

OBSTETRICS. (A. A. SMITH, M.D.)

Question 1. What is the Anteroposterior diameter (in inches) of the normal female pelvis at brim, cavity, and outlet?

Q. 2. Name the two main divisions of the female organs of generation.

Q. 3. What are the internal organs of generations?

Q. 4. Give names of the three anatomical divisions of the uterus.

Q. 5. What is the average period of gestation, in days?

Q. 6. Into how many principal stages is labor divided?

Q. 7. Give name of presentation of fetus in normal labor.

Q. 8. If called to see a case of labor and you diagnosed a case of shoulder presentation, how would you proceed to the delivery?

Q. 9. In Post-partum hemorrhage what would be your treatment?

Q. 10. In simple uterine inertia what remedies would you use to aid nature in effecting the delivery?

GYNECOLOGY. (A. A. SMITH, M.D.)

Question 1. What is Anteversion of the Uterus?

Q. 2. What is Endrometritis?

Q. 3. What is Dysmenorrhea?

Q. 4. What is Menorrhagia?

Q. 5. If called to see a patient suffering with excessive Menorrhagia, what would be your treatment?

Q. 6. What is Puerperal fever?

Q. 7. If called to see a case of well marked Puerperal fever, what would be your treatment?

Q. 8. What is Ovaritis?

Q. 9. What Antiseptics would you use in Obstetrical and Gynecological cases?

Q. 10. Explain technique of operation for removal of Ovarian cyst.

MATERIA MEDICA AND THERAPEUTICS. (W. A. O'DANIEL, M.D.)

Question 1. What are the dose, physiological action, and therapeutics of Anti-febrin?

Q. 2. What are the component parts of Dover's powders?

Q. 3. What is the dose of Atropia?

Q. 4. What class of drugs do you understand to be Errhines?

Q. 5. Give the mode of procedure in the administration of Chloroform.

Q. 6. What solvent would you give for Quinine, if you desired to give it hypodermically?

Q. 7. What amount of Iodide of Potassium could be given in each twenty-four hours in any stage of Syphilis?

Q. 8. How is a Sulpho-Carbolate prepared?

Q. 9. Give the definition of Therapeutics.

Q. 10. Write me a sample prescription.

ANATOMY. (F. M. RIDLEY, M.D.)

Question 1. (1) Name bones in Orbital Plate. (2) Classify divisions of Vertebral Column. How distinguish them. (3) Describe Humerus. Ulna.

Q. 2. (1) Give articulation of Shoulder joint. Of Hip joint.

Q. 3. (1) What muscles does the Quadriceps Extensor Femoris include? And what does its tendon contain?

Q. 4. (1) What is an Artery? (2) What Artery supplies the tissues of the Heart with blood? (3) Give branches of External Carotid Artery. (4) Describe Circle of Willis. (5) Give branches of Femoral Artery.

Q. 5. (1) What is a Vein? (2) Through what vein is arterial blood transmitted? (2) Mention some veins which have no valves.

Q. 6. (1) What are the membranes covering the Brain? (2) How is the Brain divided? (3) Give the twelve pairs of Cranial nerves. (4) Give origin, foramen of exit, and distribution of Pneumogastive nerve.

Q. 7. (1) Describe Peritoneum. (2) Describe Appendix Vermiformis. (3) What is the largest Gland in the body? Describe it. (4) Give so-called Ductless Glands. (5) Describe the Heart.

Q. 8. (1) Where are the Kidneys situated? (2) What are the Malpighian Bodies? (3) Describe the Bladder. (4) Enumerate the divisions of the Urethra.

Q. 9. (1) Describe the Uterus. (2) What is the Parovarium?

Q. 10. (1) What structures do you come in contact with in operation for Oblique Inguinal Hernia? (2) What tissues are divided in amputation at union of upper and middle third of Thigh? (3) Where is Scarpa's Triangle? Describe it. (4) Describe the Triangles of the Neck.

SURGERY. (F. M. RIDLEY, M.D.)

Question 1. What is Inflammation? Measures employed in its treatment. Define Erysipelas. Give its treatment.

Q. 2. What is a Wound? How classified under four leading heads? What is Asepsis? Explain fully Antisepsis.

Q. 3. Define Amputation. What do you understand by Point of Election? Describe fully method of Amputation at Middle Third of Thigh. Give technique of operation from preparation of patient to application of dressing.

Q. 4. What is an Abscess? What is Aneurism? What is the differential diagnosis?

Q. 5. What is a Fracture? Classify Fractures. Give symptoms of Fracture. Explain briefly process of repair. What are the cardinal points in treatment of Fractures? What is Colles's Fracture? What is Potts's Fracture?

Q. 6. What is a Dislocation? Give symptoms. Explain dislocation of Inferior Maxilla. How reduced? Explain three directions of dislocation of Humerus, at shoulder joint. We consider four distinct luxations of Femur at Hip joint: what are they, and symptoms of each? How would you reduce dislocated Patella?

Q. 7. What is Ophthalmia? What is Conjunctivitis? How treated? What is Cataract? What is Ptosis? Give treatment for Granulated lids?

Q. 8. What is Gonorrhea? How is Specific Gonorrhea treated? What is Urethral Stricture? What is Syphilis? Differential diagnosis between Chancre and Chancroid, and the treatment of each.

Q. 9. What are Hemorrhoids? What is the difference between Internal and External Hemorrhoids? Define Fissure and Fistula of Rectum, and suggest treatment of each.

Q. 10. What is Hernia? Classify Pelvic Hernias.

